# Circ_LDLR promoted the development of papillary thyroid carcinoma via regulating miR-195-5p/LIPH axis

**DOI:** 10.1186/s12935-020-01327-3

**Published:** 2020-06-15

**Authors:** Xiaolong Gui, Yan Li, Xiaobin Zhang, Ka Su, Wenlong Cao

**Affiliations:** 1 Department of Gastrointestinal & Gland Surgery, The First Affiliated Hospital of Guangxi Medical University, No. 6 Shuangyong Road, Nanning, 530021 Guangxi China; 2 Department of Pharmacy, Affiliated Tumor Hospital of Guangxi Medical University, Nanning, 530021 Guangxi China

**Keywords:** PTC, circ_LDLR, miR-195-5p, LIPH

## Abstract

**Background:**

Emerging studies have demonstrated that circular RNAs (circRNAs) are key regulators for tumorigenesis in cancers, including papillary thyroid carcinoma (PTC). In this study, we aimed to explore the effects of circ_LDLR on PTC.

**Methods:**

Quantitative real-time polymerase chain reaction (qRT-PCR) was performed to determine the levels of circ_LDLR, miR-195-5p and lipase H (LIPH). RNase R digestion assay and Actinomycin D assay were utilized to analyze the characteristics of circ_LDLR. Colony formation assay and 3-(4,5-dimethyl-2-thiazolyl)-2, 5-diphenyl-2-*H*-tetrazolium bromide (MTT) assay were conducted to evaluate cell proliferation. Western blot assay was used for the determination of protein levels. Flow cytometry analysis was applied to determine cell apoptosis. Transwell assay was performed to determine cell migration and invasion. Dual-luciferase reporter assay was used to verify the associations among circ_LDLR, miR-195-5p and LIPH. The murine xenograft model was constructed to explore the roles of circ_LDLR in vivo.

**Results:**

Compared to normal tissues and cells, circ_LDLR was upregulated in PTC tissues and cells. Silencing of circ_LDLR suppressed PTC cell colony formation, proliferation, migration and invasion and promoted apoptosis in vitro and hampered tumor growth in vivo. For mechanism investigation, circ_LDLR could regulate LIPH expression via sponging miR-195-5p. Moreover, miR-195-5p inhibition restored the effects of circ_LDLR knockdown on the malignant behaviors of PTC cells. MiR-195-5p overexpression inhibited PTC cell colony formation, proliferation, migration and invasion and facilitated apoptosis by targeting LIPH.

**Conclusion:**

Circ_LDLR knockdown decelerated PTC progression by regulating miR-195-5p/LIPH axis, which might provide a novel therapeutic target for PTC.

## Background

Papillary thyroid carcinoma (PTC) is the most common malignant tumor, accounting for about 85% of thyroid cancers [1]. Due to high degree of differentiation, slow tumor growth and good postoperative prognosis, the 10-year survival rate of PTC patients can reach more than 90% [2, 3]. Nevertheless, some PTC patients still have a poor prognosis for the aggressive characteristics, such as older age, large primary tumor, distant metastasis and lymph node metastasis [4]. Therefore, improving our knowledge on the pathogenesis of PTC and identifying novel targets for PTC therapy are of great significance.

Circular RNAs (circRNAs) are a series of non-coding RNAs (ncRNAs), which featured with covalently closed loops [5]. CircRNAs have been verified to act essential mediators in human diseases, especially cancers [6]. In PTC, several circRNAs have been identified. For instance, circRASSF2 level was raised in PTC and played an oncogenic role in PTC [7]. Circ-ITCH was lowly expressed in PTC and its overexpression repressed PTC cell growth and invasion and enhanced apoptosis [8]. These findings indicated that circRNAs played dual roles in PTC progression. As a member of circRNAs, the exact roles and molecular mechanisms of circ_LDLR (hsa_circ_0003892) in PTC have not been elucidated yet. Through analyzing Gene Expression Omnibus (GEO) dataset GSE93522 [9], we found that circ_LDLR level was elevated in PTC tumor tissues. Thus, we explored the association betweeen circ_LDLR and PTC progression in the present research.

MicroRNAs (miRNAs), ncRNAs with ~ 22 nucleotides, can modulate gene expression post-transcriptionally through interacting with the 3′ untranslated region (3′UTR) of target mRNAs [10]. Diverse miRNAs, such as miR-146b [11], miR-34a [12] and miR-204-5p [13] have been proved to function as vital regulators in PTC development. Moreover, couples of reports have shown that miR-195-5p can exert its tumor-suppressive role in different types of tumor, such as prostate cancer [14], osteosarcoma [15] and cervical cancer [16]. A former study showed that miR-195 functioned as a tumor suppressor in PTC by targeting CCND1 and FGF2 [17]. Nonetheless, the precise roles of miR-195-5p in PTC are not entirely understood.

Lipase H (LIPH) is a secretase that hydrolyzes phosphatidic acid into free fatty acids and lysophosphatidic acid which is involved in cellular process [18, 19]. Recently, the effects of LIPH on cancers are increasingly being investigated. For instance, Ishimine et al. suggested that LIPH level was increased in esophageal adenocarcinomas and related to overall survival of patients [20]. Cui et al. declared that LIPH was associated with the tumor size, lymph node metastasis, and distant metastasis of breast cancer [21]. Moreover, a previous study demonstrated that LIPH could aggravate PTC development [22]. However, whether LIPH could be targeted by miR-195-5p remains unknown.

In the research, we checked the expression patterns of circ_LDLR, miR-195-5p and LIPH in PTC tussues and cells. Moreover, the biological roles and potential mechanisms of circ_LDLR in PTC were investigated through function and mechanism analysis.

## Materials and methods

### Specimens collection

60 pairs of PTC tissues and adjacent normal thyroid tissues were obtained from PTC patients who underwent surgery at the First Affiliated Hospital of Guangxi Medical University and frozen at − 80 °C until use. The patients enrolled in our study did not receive any treatment prior to this surgical procedure. The clinicopathological data of the PTC patients were showed in Table 1. The procedures were permitted by the Ethics Committee of the First Affiliated Hospital of Guangxi Medical University and written informed consents were provided by the patients.

**Table 1:** Clinicopathological data of the PTC patients

Parameters	Groups	Numbers (n = 60)
Age	≤ 40	41
	> 40	19
Gender	Male	25
	Female	35
Multifocality	No	26
	Yes	34
Lymphatic metastasis	No	27
	Yes	33
TNM stage	I + II	39
	II + III	21

### Cell culture

Human thyroid cell line Nthy-ori3-1 was purchased from BNBIO (BNCC353368; Beijing, China), PTC cell lines K-1 and SW1736 were bought from Shanghai Institute of Cell Biology (0292, C10311; Shanghai, China), PTC cell line TPC-1 was purchased from COBIOER (CBP60257; Nanjing, China) and PTC cell line SW579 was obtained from Procell (CL-0224; Wuhan, China). All cells were maintained in a humidified incubator (95% air/5% CO_2_) at 37 °C in RPMI1640 (Invitrogen, Carlsbad, CA, USA) containing 1% penicillin–streptomycin (Solarbio, Beijing, China) and 10% fetal bovine serum (FBS; Solarbio).

### Quantitative real-time polymerase chain reaction (qRT-PCR)

Total RNA was isolated using TRIzol reagent (Beyotime, Shanghai, China) and quantified on a NanoDrop 2000c spectrophotometer (Thermo Fisher Scientific, Waltham, MA, USA). Reverse transcription experiment was conducted using PrimeScript™ RT reagent Kit (Takara, Dalian, China) or TaqMan MicroRNA Reverse Transcription Kit (Applied Biosystems, Foster City, CA, USA). Next, the BeyoFast™ SYBR Green qPCR Mix (Beyotime) was utilized to conduct qRT-PCR. The relative expression was estimated using the 2^−ΔΔCt^ method with U6 or glyceraldehyde 3-phosphate dehydrogenase (GAPDH) as an internal reference. The sequences of primers were: circ_LDLR: (F: 5′-GTGAGGGCTCTGTCCATTGT-3′ and R: 5′-GGTGGTCCTCTCACACCAGT-3′); LDLR: (F: 5′-CAATGTCTCACCAAGCTCTG-3′ and R: 5′-TCTGTCTCGAGGGGTAGCTG-3′); miR-195-5p: (F: 5′-GGGGTAGCAGCACAGAAAT-3′ and R: 5′-TCCAGTGCGTGTCGTGGA-3′); LIPH: (F: 5′-CTGATGCTCTACACAAGGA-3′ and R: 5′-ATGGACAATGAAGGTGGTT-3′); GALNT7: (F: 5′-GGTACCATGGCCTCATGTTG-3′ and R: 5′-GCCACCACACTGCCATATCT-3′); PSD3: (F: 5′-GCTCTGTACAACTCAATCAAGAATG-3′ and R: 5′-CCAATACGACTGATGGTCTTTG-3′); ITGA2: (F: 5′-CCTACAATGTTGGTCTCCCAGA-3′ and R: 5′-AGTAACCAGTTGCCTTTTGGATT-3′); GAPDH: (F: 5′-CTGGGCTACACTGAGCACC-3′ and R: 5′-AAGTGGTCGTTGAGGGCAATG-3′); U6: (F: 5′-TTATGGGTCCTAGCCTGAC-3′ and R: 5′-CACTATTGCGGGTCTGC-3′).

### RNase R digestion assay

10 μg total RNA was incubated with RNase R (20 mg/mL; Solarbio) for 20 min at 37 °C. Total RNA untreated with RNase was used as a control. Then qRT-PCR was carried out to measure the levels of circ_LDLR and LDLR mRNA.

### Actinomycin D assay

After TPC-1 and SW579 cells (5 × 10^4^ cells/well) were seeded into 24-well plates and incubated overnight, 2 μg/mL Actinomycin D (Abcam, Cambridge, MA, USA) was added to block transcription at indicated time points. Next, qRT-PCR was conducted to examine the expression of circ_LDLR and LDLR mRNA.

### Cell transfection

The small interfering RNA targeting circ_LDLR (si-circ_LDLR; 5′-CCTCCCCATCGGACAAAGTAT-3′), mimics of miR-195-5p (miR-195-5p; 5′-UAGCAGCACAGAAAUAUUGGC-3′), inhibitors of miR-195-5p (anti-miR-195-5p; 5′- GCCAAUAUUUCUGUGCUGCUA-3′), short hairpin RNA against circ_LDLR (sh-circ_LDLR; 5′-GTCCTCCCCATCGGACAAAGT-3′) and their controls were bought from Sangon (Shanghai, China). The overexpression vector of LIPH (LIPH) was constructed by introducing LIPH sequences (NM_139248.3) into pcDNA3.1 vector (Invitrogen) and blank pcDNA3.1 vector was used as control. Lipofectamine 2000 (Invitrogen) was utilized for cell transfection.

### Colony formation assay

After relevant transfection, PTC cells were seeded into 6-well plates (300 cells/well). The culture medium was changed every 3 days. 10 days later, the colonies were fixed with 4% paraformaldehyde (Sangon) and stained with 0.2% crystal violet (Solarbio). Finally, the colonies with a minimum of 50 cells were counted and photographed. The number of cell colonies was counted in five random fields.

### 3-(4,5-Dimethyl-2-thiazolyl)-2, 5-diphenyl-2-*H*-tetrazolium bromide (MTT) assay

The proliferation of PTC cells was determined through MTT assay. Transfected PTC cells (2 × 10^4^ cells/well) were collected and plated into 96-well plates and kept overnight. Then, 20 μL MTT (5 mg/mL; Solarbio) was added into the well at indicated time points and cultured for another 4 h. Afterward, the formazan crystals were dissolved with 150 µL dimethyl sulfoxide (DMSO; Solarbio) followed by determination of OD value at 490 nm with a microplate reader (Bio-Rad Laboratories, Hercules, CA, USA).

### Western blot assay

Total protein was isolated with RIPA buffer (Beyotime) and determined with a BCA protein assay kit (Beyotime). Then the extracts were separated by sodium dodecyl sulfonate-polyacrylamide gel (Solarbio) and transferred to polyvinylidene difluoride membranes (Millipore, Billerica, MA, USA). Next, the membranes were blocked in 5% slim milk for 1 h at room temperature and incubated overnight at 4 °C with primary antibodies: Ki67 (ab16667; 1:1000; Abcam), Twist1 (ab49254; 1:500; Abcam), E-cadherin (ab231303; 1:1000; Abcam), LIPH (ab192615; 1:5000; Abcam), GALNT7 (ab113743; 1:1000; Abcam), PSD3 (ab62194; 1:2000; Abcam), ITGA2 (ab133557; 1:20,000; Abcam) or GAPDH (ab181602; 1:10,000; Abcam). Afterward, the samples were subjected to secondary antibody (ab205719; 1:10,000; Abcam) for 2 h at room temperature. At last, the protein bands were determined using ECL western blot kit (Beyotime) and analyzed by ImageJ software.

### Flow cytometry analysis

Annexin V-fluorescein isothiocyanate (FITC)/propidium iodide (PI) Apoptosis Detection Kit (Beyotime) was used to determine cell apoptosis. Briefly, transfected PTC cells (1 × 10^6^ cells) were harvested and suspended in binding buffer. Then Annexin-FITC and PI were added to stain cells in the dark. 15 min later, the apoptotic cells were analyzed by FACScan^®^ flow cytometry (BD Biosciences, San Jose, CA, USA).

### Transwell assay

Transwell insert chambers (Scipu001412; Corning, Corning, NY, USA) pre-coated with (for cell invasion assay) or without (for cell migration assay) Matrigel (Corning) were utilized to evaluate the invasion and migration of PTC cells. In brief, about 1 × 10^6^ transfected cells suspended in 300 μL serum-free RPMI1640 (Invitrogen) were added into the upper chamber and 500 μL culture medium was added into the bottom chamber. After 24 h, migrated/invaded cells were fixed with 4% paraformaldehyde (Sangon), stained with crystal violet (Solarbio) and then observed with an inverted microscope (Olympus, Tokyo, Japan). Photographs of 5 randomly selected fields were taken and migrated/invaded cell number per field = the total count of five high power fields/5.

### Dual-luciferase reporter assay

The fragments of circ_LDLR and LIPH 3′UTR containing the predicted binding sites of wild-type or mutant miR-195-5p were inserted into pmirGLO plasmid (Promega, Madison, WI, USA) to construct the luciferase reporter vectors circ_LDLR-wt, circ_LDLR-mut, LIPH-wt and LIPH-mut, respectively. Then PTC cells were seeded into 24-well plates (1 × 10^5^ cells/well) and transfected with miR-NC or miR-195-5p together with corresponding luciferase reporter vector. After 48 h, Dual-Luciferase Reporter Assay Kit (Promega) was used to measure the luciferase activity.

### Murine xenograft model

A total of 14 female BALB/c nude mice (4–6 weeks old; Shanghai SLAC Laboratory Animals Co., Ltd, Shanghai, China) were randomly divided into 2 groups (N = 7/group). Lentivirus-mediated sh-circ_LDLR or sh-NC was transfected into TPC-1 cells. Then a total of 2 × 10^6^ transfected cells in 200 μL PBS were subcutaneously injected into the mice. Tumor volume was measured weekly and calculated using the formula: (length × width^2^)/2. After 4 weeks, the mice were euthanized and tumors were weighted. The collected tumor samples were preserved at − 80 °C.

### Statistical analysis

The experiments were repeated at least three times. The obtained data were analyzed by GraphPad Prism 7 (GraphPad Inc., La Jolla, CA, USA) and expressed as mean ± standard deviation (SD). Differences were estimated by Student’s *t*-test or one-way analysis of variance (ANOVA). The correlations among the expression of circ_LDLR, miR-195-5p and LIPH mRNA in PTC tissues were analyzed by Spearman’s correlation coefficient analysis. It was deemed as statistically significant if *P *< 0.05.

## Results

### Circ_LDLR was highly expressed in PTC tissues and cells

**Fig. 1:**
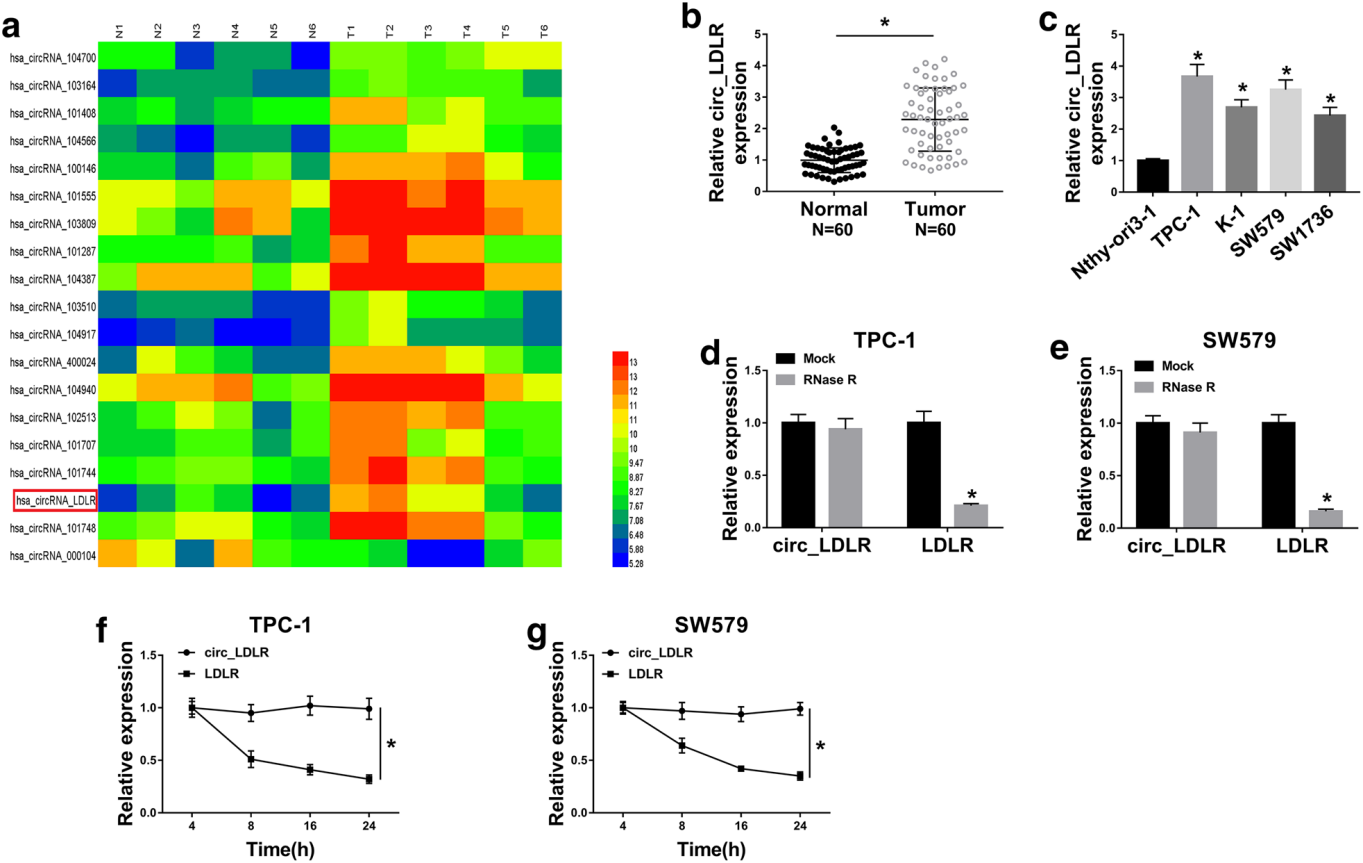
Circ_LDLR level was elevated in PTC tissues and cells. **a** Circ_LDLR was upregulated in PTC according to the analysis of GEO dataset GSE93522. **b**, **c** The expression level of circ_LDLR in PTC tissues (N = 60), cells and corresponding normal tissues and cells was determined using qRT-PCR (Student’s *t*-test). **d**, **e** The expression levels of circ_LDLR and LDLR were measured by qRT-PCR after treatment with or without RNase R in TPC-1 and SW579 cells. **f**, **g** The expression levels of circ_LDLR and LDLR were examined using qRT-PCR after treatment with Actinomycin D at indicated time points in TPC-1 and SW579 cells. *P < 0.05. Each bar represents mean ± SD

Through analyzing GEO dataset GSE93522, circ_LDLR was found to be upregulated in PTC samples (Fig. 1a). Next, the expression level of circ_LDLR in 60 pairs of PTC tissues and corresponding normal tissues was determined by qRT-PCR. The data showed that circ_LDLR was notably elevated in PTC tissues in comparison with normal tissues (Fig. 1b). Moreover, circ_LDLR was markedly highly expressed in PTC cells (TPC-1, K-1, SW579 and SW1736) when compared to Nthy-ori3-1 cells (Fig. 1c). In view of the expression of circ_LDLR in TPC-1 and SW579 cells was higher than in K-1 and SW1736 cells, these two cell lines were used for the subsequent experiments. Afterward, the circular characteristics of circ_LDLR were investigated using RNase R and Actinomycin D assays. RNase R assay indicated that circ_LDLR was resistant to RNase R, while linear LDLR mRNA was distinctly digested by RNase R (Fig. 1d, e). The results of Actinomycin D assay showed that circ_LDLR had a longer half-life compared to linear LDLR in both TPC-1 and SW579 cells (Fig. 1f, g). Collectively, circ_LDLR was a circular and stable transcript, and upregulated in PTC.

### Knockdown of circ_LDLR suppressed cell colony formation, proliferation, migration and invasion and promoted apoptosis in PTC cells

**Fig. 2:**
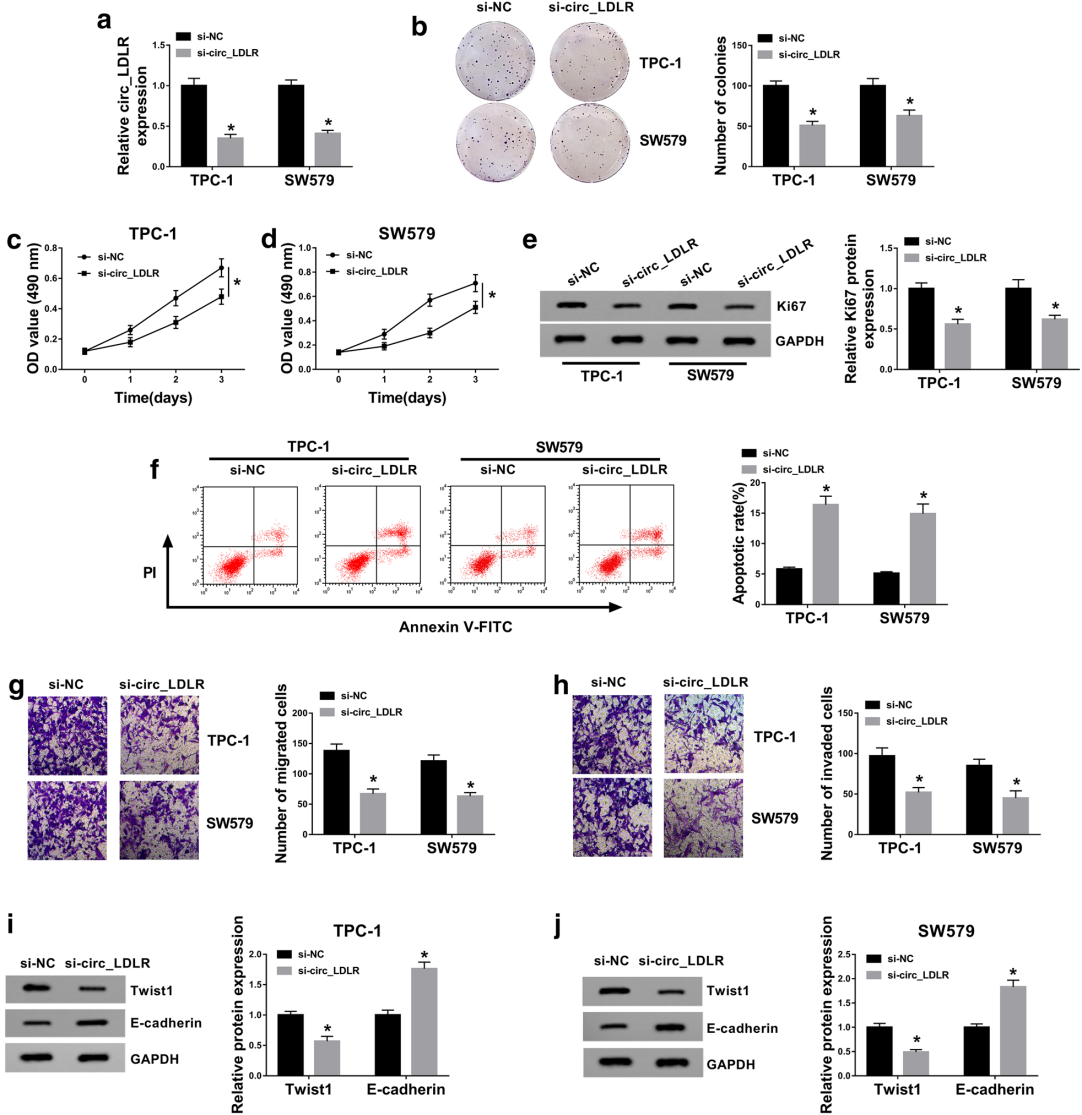
Silencing of circ_LDLR inhibited PTC cell colony formation, proliferation, migration and invasion and induced cell apoptosis. TPC-1 and SW579 cells were transfected with si-NC or si-circ_LDLR. **a** The expression of circ_LDLR in TPC-1 and SW579 cells was examined using qRT-PCR. **b** The colony formation of TPC-1 and SW579 cells was assessed by colony formation assay. **c**, **d** The proliferation of TPC-1 and SW579 cells was evaluated using MTT assay. **e** The protein level of Ki67 in TPC-1 and SW579 cells was measured by western blot assay. **f** The apoptosis of TPC-1 and SW579 cells was analyzed by flow cytometry analysis. **g**, **h** The migration and invasion of TPC-1 and SW579 cells were evaluated by transwell assay. **i**, **j** The protein levels of Twist1 and E-cadherin in TPC-1 and SW579 cells were measured through western blot assay. *P < 0.05. Each bar represents mean ± SD

In order to explore the precise roles of circ_LDLR in PTC, si-circ_LDLR was transfected into TPC-1 and SW579 cells to downregulate circ_LDLR expression. The transfection efficiency was determined using qRT-PCR. The data showed that si-circ-LDLR transfection markedly reduced the expression of circ_LDLR in TPC-1 and SW579 cells, indicating that si-circ_LDLR was successfully transfected into the TPC-1 and SW579 cells (Fig. 2a). Moreover, we tested the impact of circ_LDLR knockdown on the level of linear LDLR in TPC-1 and SW579 cells and found that there was no significant change in linear LDLR mRNA level (Additional file 1: Fig. S1a and b). Colony formation assay indicated that circ_LDLR silencing conspicuously suppressed the colony formation ability of TPC-1 and SW579 cells compared to control groups (Fig. 2b). Our results of MTT assay displayed that there was an inhibition in the proliferation of TPC-1 and SW579 cells following the downregulation of circ_LDLR in reference to control groups (Fig. 2c, d). Meanwhile, we found that the protein level of Ki67 was distinctly decreased in TPC-1 and SW579 cells after circ_LDLR knockdown (Fig. 2e). Flow cytometry analysis showed that circ_LDLR silencing evidently promoted cell apoptosis in TPC-1 and SW579 cells (Fig. 2f). The results of transwell assay indicated that circ_LDLR interference drastically repressed the migration and invasion of TPC-1 and SW579 cells when compared to control groups (Fig. 2g, h). In addition, we measured the levels of epithelial-mesenchymal transition (EMT)-related proteins (Twist1 and E-cadherin) by western blot assay. The data showed that circ_LDLR knockdown led to a marked decrease in Twist1 level and a remarkable increase in E-cadherin level in both TPC-1 and SW579 cells (Fig. 2i, j). Furthermore, we successfully transfected the overexpression vector of circ_LDLR intoTPC-1 and SW579 cells (Additional file 2: Fig. S2a). We demonstrated that circ_LDLR overexpression exhibited the opposite results in cell colony formation, proliferation, apoptosis, migration and invasion as well as the protein levels of Ki67, Twist1 and E-cadherin in TPC-1 and SW579 cells (Additional file 2: Fig. S2f–m). All these data indicated that the promotional roles of circ_LDLR in the malignant biological behaviors of PTC cells.

### Circ_LDLR negatively regulated miR-195-5p expression by directly targeting

**Fig. 3:**
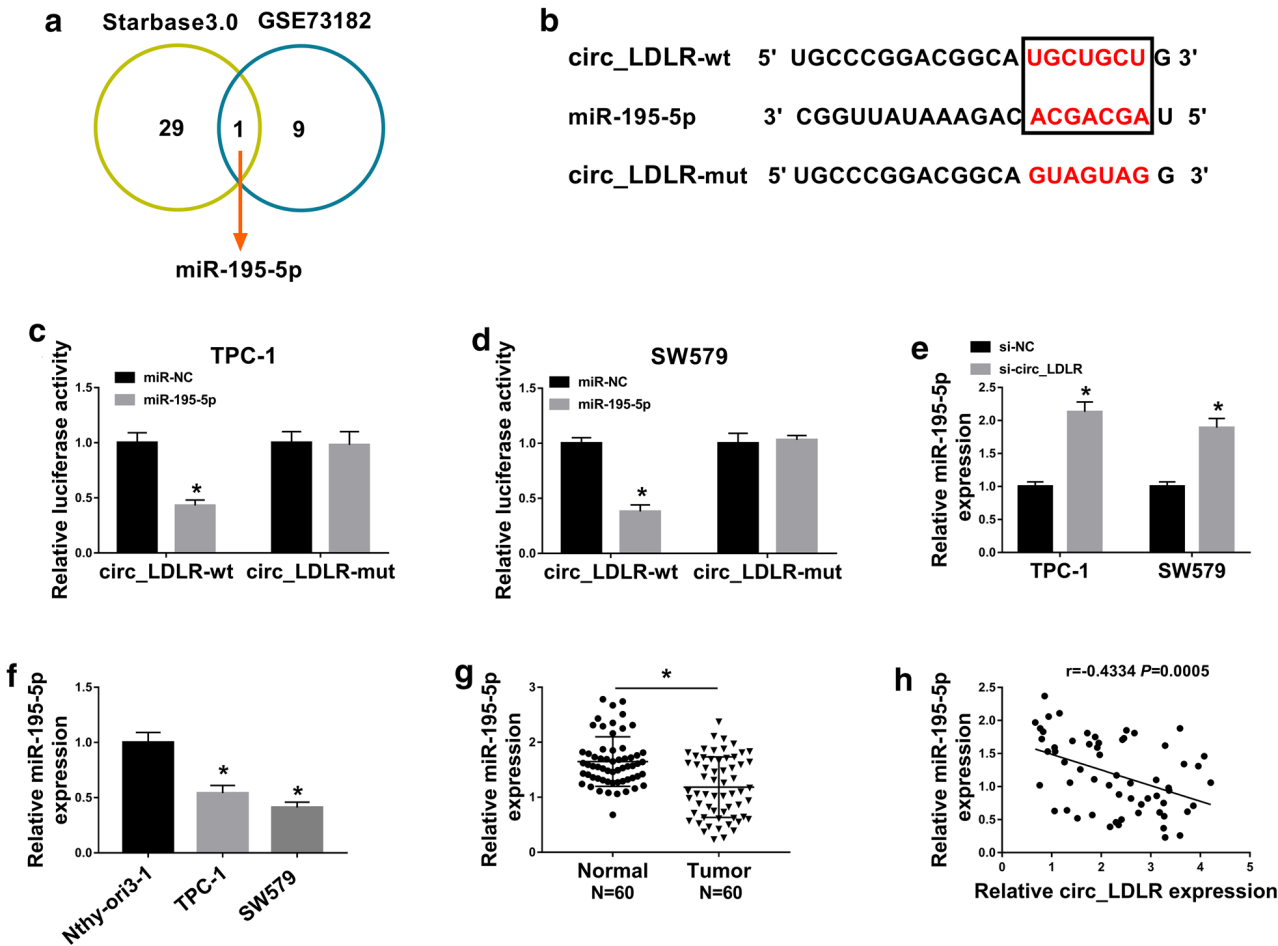
MiR-195-5p was a direct target of circ_LDLR in PTC cells. **a** MiR-195-5p was predicted to be a target of circ_LDLR. **b** The potential binding sequences between circ_LDLR and miR-195-5p were presented. **c**, **d** The luciferase activity in TPC-1 and SW579 cells co-transfected with circ_LDLR-wt or circ_LDLR-mut and miR-NC or miR-195-5p was measured by dual-luciferase reporter assay. **e** The expression of miR-195-5p in TPC-1 and SW579 cells transfected with si-NC or si-circ_LDLR was examined by qRT-PCR assay. **f**, **g** The expression of miR-195-5p in PTC cells and tissues (N = 60) was determined using qRT-PCR assay (Student’s *t*-test). **h** The correlation between circ_LDLR and miR-195-5p in PTC tissues was analyzed by Spearman’s correlation coefficient analysis. *P < 0.05. Each bar represents mean ± SD

To explore the potential mechanism of circ_LDLR in regulating PTC progression, we used online software StarBase 3.0 to search the potential target miRNAs of circ_LDLR. We found there were 29 miRNAs might be the targets of circ_LDLR. Moreover, through analyzing GEO dataset GSE73182 [23], we found 9 miRNAs were downregulated in PTC. Among them, miR-195-5p was predicted to be a target of circ_LDLR and miR-195-5p level was downregulated in PTC (Fig. 3a), so we chose miR-195-5p as our research object. The predicted binding sites between circ_LDLR and miR-195-5p were shown in Fig. 3b. Then dual-luciferase reporter assay was carried out to verify this prediction. The data showed that the co-transfection of circ_LDLR-wt and miR-195-5p evidently suppressed the luciferase activity in TPC-1 and SW579 cells compared to circ_LDLR-wt and miR-NC co-transfected groups, whereas the luciferase activity was not affected in circ_LDLR-mut groups (Fig. 3c, d). Moreover, we observed that circ_LDLR knockdown drastically elevated the expression of miR-195-5p, while circ_LDLR overexpression distinctly reduced the expression of miR-195-5p in both TPC-1 and SW579 cells (Fig. 3e and Additional file 2: Fig. S2b ). Expectably, miR-195-5p was lowly expressed in PTC cells and tissues (N = 60) in reference to corresponding normal cells and tissues (Fig. 3f, g). As analyzed by Spearman’s correlation coefficient analysis, miR-195-5p expression was inversely correlated with circ_LDLR expression in PTC tissues (Fig. 3h). To sum up, circ_LDLR negatively modulated miR-195-5p expression via direct interaction in PTC cells.

### MiR-195-5p inhibition restored the effects of circ_LDLR silencing on cell colony formation, proliferation, apoptosis, migration and invasion in PTC cells

**Fig. 4:**
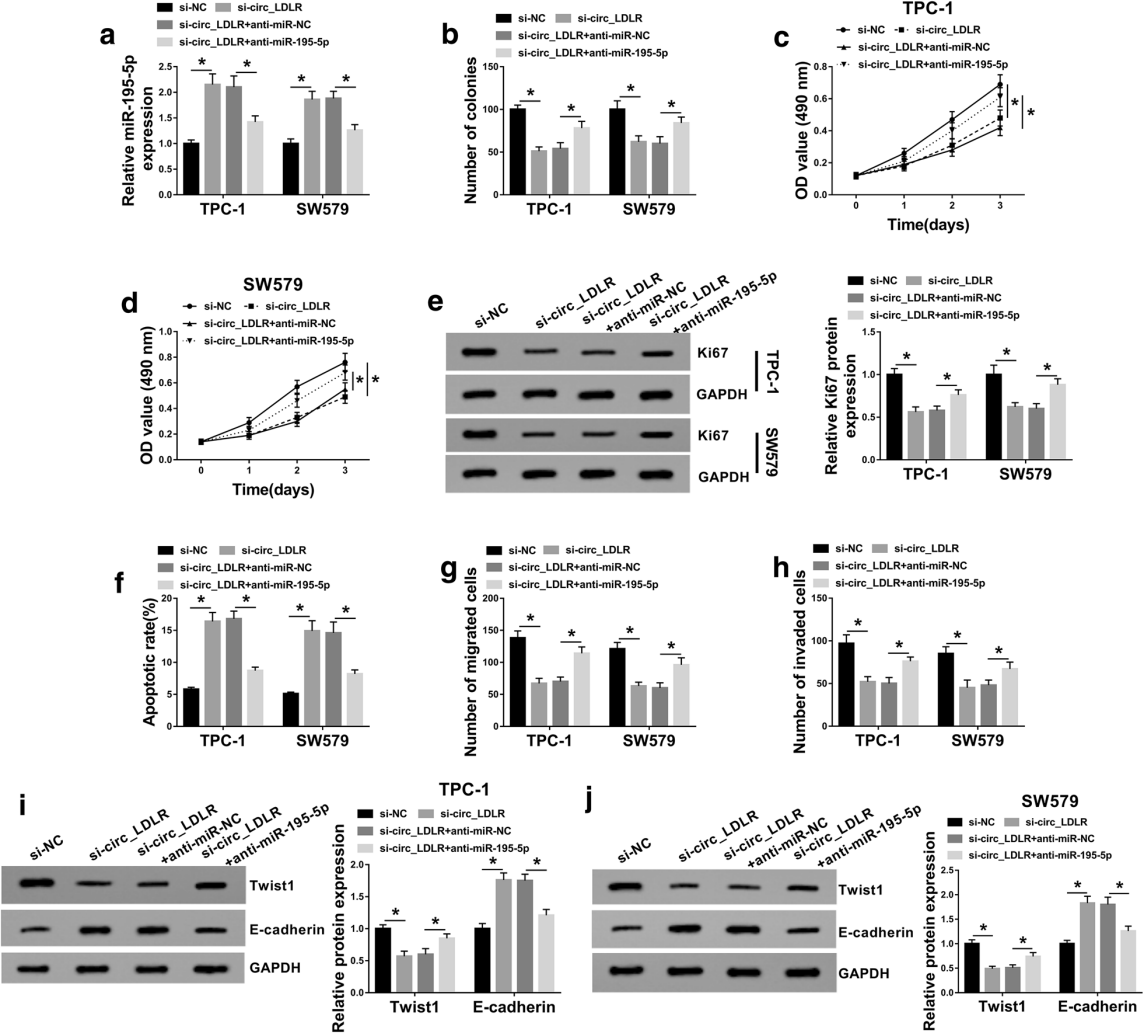
MiR-195-5p inhibition reversed the inhibitory effect of circ_LDLR knockdown on PTC cell progression. TPC-1 and SW579 cells were treated with si-NC, si-circ_LDLR, si-circ_LDLR + anti-miR-NC or si-circ_LDLR + anti-miR-195-5p. **a** MiR-195-5p expression level in TPC-1 and SW579 cells was measured by qRT-PCR. **b** Cell colony formation ability in TPC-1 and SW579 cells was assessed by colony formation assay. **c**, **d** Cell proliferation in TPC-1 and SW579 cells was evaluated by MTT assay. **e** The protein level of Ki67 in TPC-1 and SW579 cells was examined using western blot assay. **f** Cell apoptosis in TPC-1 and SW579 cells was analyzed using flow cytometry analysis. **g**, **h** Cell migration and invasion in TPC-1 and SW579 cells were determined by transwell assay. **i**, **j** The protein levels of Twist1 and E-cadherin in TPC-1 and SW579 cells were detected through western blot assay. *P < 0.05. Each bar represents mean ± SD

As shown in Additional file 3: Fig. S3a–j, the transfection of anti-miR-195-5p effectively promoted the colony formation, proliferation, migration and invasion and suppressed the apoptosis of TPC-1 and SW579 cells; moreover, the levels of proliferation-related protein Ki67 and EMT-related marker Twist1 was increased and the EMT-related marker E-cadherin level was decreased in TPC-1 and SW579 cells, indicating that miR-195-3p knockdown played a positive role in PTC  cell progression. To determine whether circ_LDLR could regulate PTC cell progression by targeting miR-195-5p, TPC-1 and SW579 cells were divided into 4 groups: si-NC, si-circ_LDLR, si-circ_LDLR + anti-miR-NC and si-circ_LDLR + anti-miR-195-5p. As presented in Fig. 4a, the elevation of miR-195-5p caused by circ_LDLR silencing was abated following the transfection of anti-miR-195-5p in both TPC-1 and SW579 cells. As demonstrated by colony formation assay and MTT assay, the inhibitory effects on cell colony formation and cell proliferation mediated by circ_LDLR knockdown were effectively weakened by miR-195-5p inhibition in TPC-1 and SW579 cells (Fig. 4b–d). In addition, circ_LDLR knockdown reduced the protein level of Ki67 in TPC-1 and SW579 cells, while the inhibition of miR-195-5p partially reversed the reduction (Fig. 4e). Moreover, our results showed that the promotional role in cell apoptosis and the suppressive roles in cell migration and invasion mediated by circ_LDLR silencing were all ameliorated following miR-195-5p deficiency in both TPC-1 and SW579 cells (Fig. 4f–h). By using western blot assay, we observed that circ_LDLR interference markedly decreased Twist1 level and increased E-cadherin level in TPC-1 and SW579 cells, while miR-195-5p inhibition abrogated the impacts (Fig. 4i, j). Taken together, circ_LDLR promoted PTC cell progression through targeting miR-195-5p.

### LIPH was a direct target gene of miR-195-5p

**Fig. 5:**
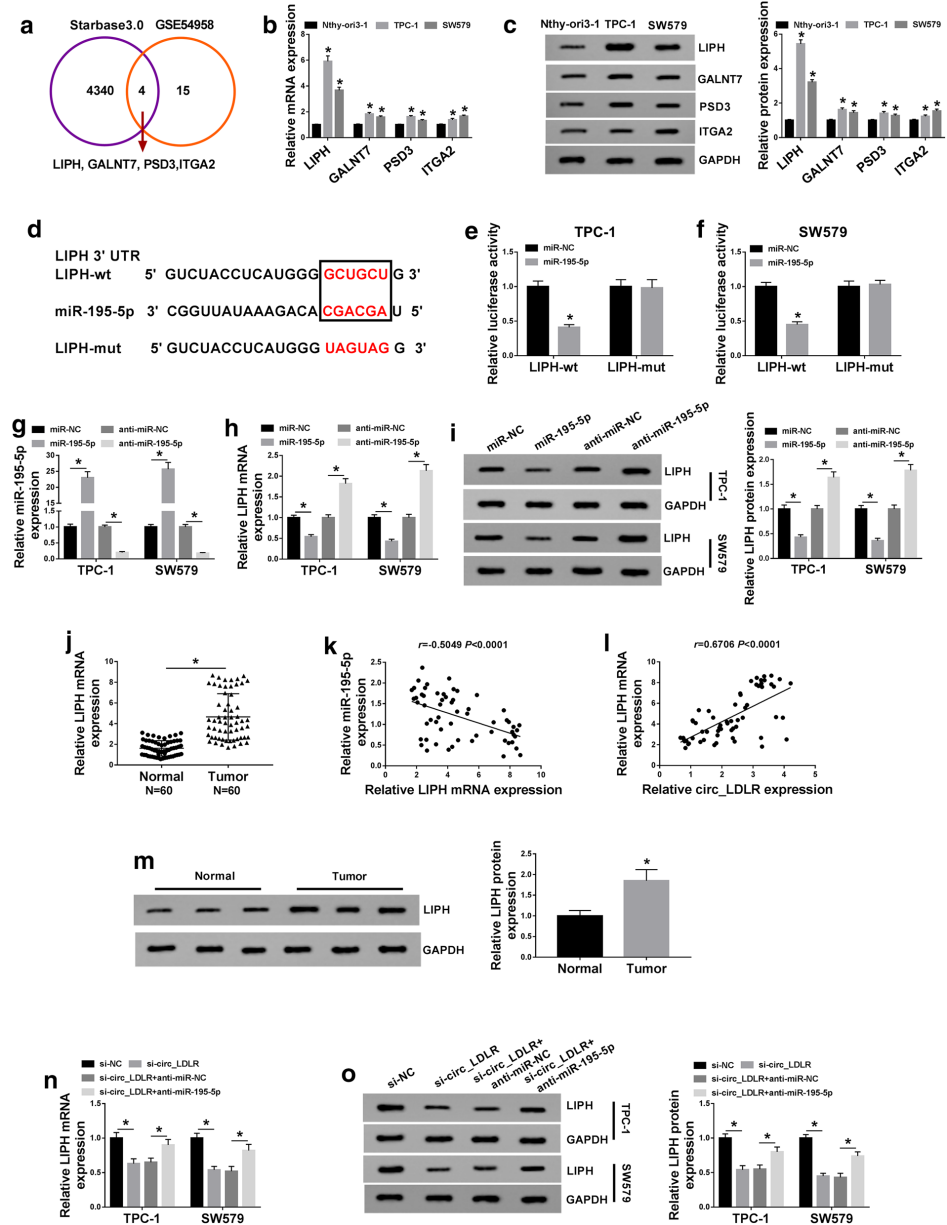
LIPH was identified as a target gene of miR-195-5p in PTC cells. **a** The targeted genes of miR-195-5p analyzed by StarBase 3.0 and GEO dataset GSE54958. **b**, **c** The mRNA and protein levels of LIPH, GALNT7, PSD3 and ITGA2 in Nthy-ori3-1, TPC-1 and SW579 cells were measured by qRT-PCR assay and western blot assay. **d** The potential biding sites between the 3′UTR of LIPH and miR-195-5p were presented. **e**, **f** The targeting relationship between miR-195-5p and LIPH was investigated by dual-luciferase reporter assay. **g**–**i** MiR-NC, miR-195-5p, anti-miR-NC or anti-miR-195-5p was transfected into TPC-1 and SW579 cells and then the levels of miR-195-5p, LIPH mRNA and LIPH protein were detected using qRT-PCR assay or western blot assay. **j** The mRNA expression of LIPH in PTC and normal tissues (N = 60) was determined using qRT-PCR (Student’s *t*-test).. **k**, **l** The correlations between miR-195-5p and LIPH mRNA, as well as LIPH mRNA and circ_LDLR in PTC tissues were analyzed by Spearman’s correlation coefficient analysis. **m** The protein level of LIPH in PTC tissues and normal tissues was measured using western blot assay. **n**, **o** TPC-1 and SW579 cells were transfected with si-NC, si-circ_LDLR, si-circ_LDLR + anti-miR-NC or si-circ_LDLR + anti-miR-195-5p and then the mRNA and protein levels of LIPH were detected through qRT-PCR assay and western blot assay, respectively. *P < 0.05. Each bar represents mean ± SD

In order to further explore the mechanism of circ_LDLR in PTC, we found 4340 target genes of miR-195-5p through analyzing online tool StarBase 3.0. Then we used GEO dataset GSE54958 [24] and 15 genes expressed in PTC was found to be 5 times higher than controls. Among them, 4 target genes (LIPH, GALNT7, PSD3 and ITGA) of miR-195-5p were up-regulated in PTC (Fig. 5a). Next, the mRNA and protein levels of these 4 genes in TPC-1 and SW579 cells were determined by qRT-PCR assay and western blot assay, respectively. Our data showed that the mRNA and protein levels of these 4 genes were obviously upregulated in TPC-1 and SW579 cells compared to Nthy-ori3-1 cells (Fig. 5b, c). Subsequently, we selected LIPH as our experimental subject for its higher expression in PTC cells than in the other genes. The potential complementary sequences between miR-195-5p and LIPH were shown in Fig. 5d. The results of dual-luciferase reporter assay indicated that there was an inhibition in the luciferase activity in LIPH-wt and miR-195-5p co-transfected TPC-1 and SW579 cells compared to LIPH-wt and miR-NC co-transfected groups, but no change was observed in LIPH-mut groups, further confirming the combination between miR-195-5p and LIPH (Fig. 5e, f). Additionally, miR-195-5p, anti-miR-195-5p or matched control was transfected into TPC-1 and SW579 cells. We found that miR-195-5p transfection apparently elevated the expression of miR-195-5p and significantly reduced the mRNA and protein levels of LIPH in TPC-1 and SW579 cells, whereas anti-miR-195-5p transfection showed the opposite results (Fig. 5g–i). Compared to normal tissues, the mRNA and protein levels of LIPH were increased in PTC tissues (N = 60) (Fig. 5j, l). Furthermore, we found that LIPH mRNA was negatively correlated with miR-195-5p expression and positively correlated with circ_LDLR expression in PTC tissues (Fig. 5k, l). In addition, circ_LDLR overepxression markedly enhanced the mRNA and protein levels of LIPH in TPC-1 and SW579 cells compared to control groups (Additional file 2: Fig. S2c, d). As expected, LIPH protein level was upregulated in PTC tissues (N = 60) compared to normal tissues (Fig. 5m). Thereafter, we investigated the relationship among circ_LDLR, miR-195-5p and LIPH in PTC cells. Our data showed that circ_LDLR knockdown remarkably reduced the expression levels of LIPH mRNA and protein in TPC-1 and SW579 cells, while the impacts were abrogated by miR-195-5p inhibition (Fig. 5n, o). All the results illustrated that circ_LDLR positively regulated LIPH expression through targeting miR-195-5p in PTC cells.

### MiR-195-5p overexpression restrained cell colony formation, cell proliferation, migration and invasion and induced apoptosis by targeting LIPH in PTC cells

**Fig. 6:**
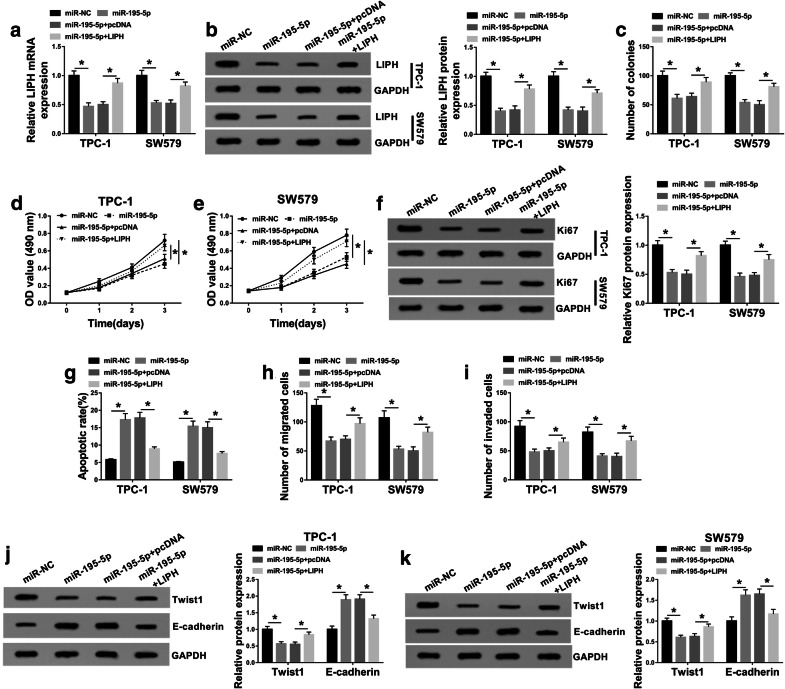
LIPH overexpression rescued the effect of miR-195-5p on PTC cell progression. TPC-1 and SW579 cells were treated with miR-NC, miR-195-5p, miR-195-5p + pcDNA or miR-195-5p + LIPH. **a**, **b** LIPH mRNA and protein levels in TPC-1 and SW579 cells were examined by qRT-PCR assay and western blot assay. **c** The colony formation of TPC-1 and SW579 cells was determined by colony formation assay. **d**, **e** The proliferation of TPC-1 and SW579 cells was evaluated by MTT assay. **f** The protein level of Ki67 was measured by western blot assay. **g** The apoptosis of TPC-1 and SW579 cells was assessed using flow cytometry analysis. **h**, **i** The migration and invasion of TPC-1 and SW579 cells were measured through transwell assay. **j**, **k** The protein levels of Twist1 and E-cadherin in TPC-1 and SW579 cells were detected via western blot assay. *P < 0.05. Each bar represents mean ± SD

Based on the above experimental results, we wondered whether miR-195-5p could participate in regulating PTC development by interacting with LIPH. To explore it, we assigned TPC-1 and SW579 cells into 4 groups: miR-NC, miR-195-5p, miR-195-5p + pcDNA and miR-195-5p + LIPH. As we observed in Fig. 6a, b, the elevation of miR-195-5p notably suppressed the mRNA and protein levels of LIPH in TPC-1 and SW579 cells, whereas the effects were rescued by LIPH overexpression. The results of colony formation and MTT assay indicated that the colony formation and proliferation abilities of TPC-1 and SW579 cells were repressed by miR-195-5p overexpression, while LIPH upregulation abolished the influences (Fig. 6c–e). Western blot assay showed that LIPH overexpression overturned the suppressive effect of miR-195-5p on Ki67 protein level in TPC-1 and SW579 cells (Fig. 6f). As demonstrated by flow cytometry analysis, transwell assay and western blot assay, miR-195-5p overexpression promoted cell apoptosis and E-cadherin level and impeded cell migration, invasion and Twist1 level in TPC-1 and SW579 cells, whereas the upregulation of LIPH effectively restored the impacts (Fig. 6g–k). These results indicated that miR-195-5p slowed PTC cell progression by interacting with LIPH.

### Silencing of circ_LDLR blocked tumorigenesis of PTC in vivo

**Fig. 7:**
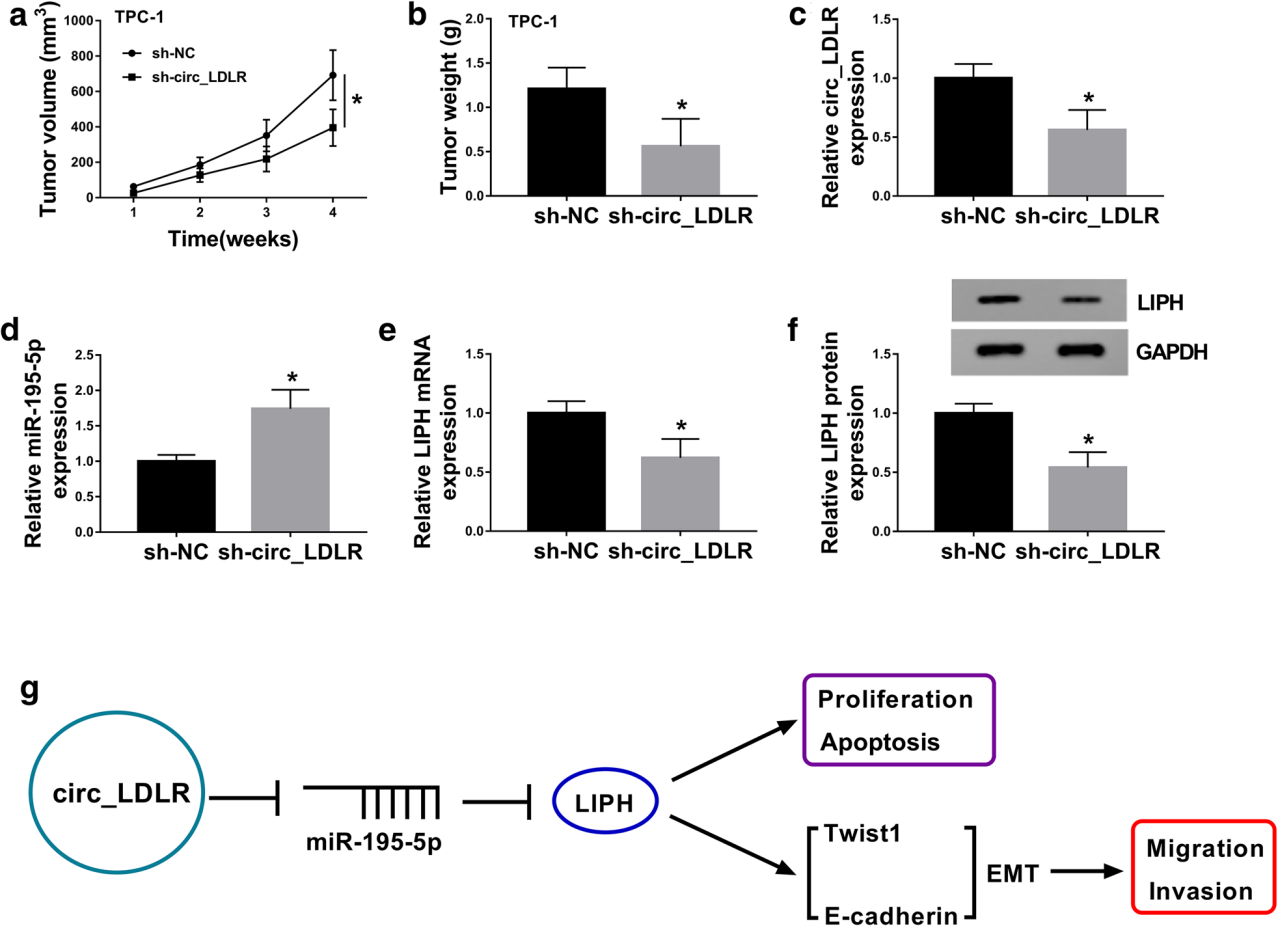
Circ_LDLR knockdown suppressed tumor growth in vivo. **a** Tumor volume was monitored every week. **b** Tumors were collected and weighted after 4 weeks. **c**–**e** The levels of circ_LDLR, miR-195-5p and LIPH mRNA in the collected tumors were determined using qRT-PCR. **f** The protein level of LIPH in the collected tumors was examined via western blot assay. **g** The schematica representation of circ_LDLR/miR-195-5p/LIPH axis in the modulation of PTC progression. *P < 0.05. Each bar represents mean ± SD

In order to reveal the functional role of circ_LDLR in PTC in vivo, sh-circ_LDLR or sh-NC transfected TPC-1 cells were injected into nude mice. As we observed in Fig. 7a, b, tumor volume and weight were notably restrained in sh-circ_LDLR groups compared to sh-NC groups. Then the levels of circ_LDLR, miR-195-5p, LIPH mRNA and LIPH protein were measured using qRT-PCR assay or western blot assay. The data showed that the levels of circ_LDLR, LIPH mRNA and LIPH protein were downregulated and miR-195-5p was upregulated in the tumors collected from sh-LDLR groups compared to sh-NC groups (Fig. 7c–f). The data suggested that circ_LDLR knockdown impeded tumor growth in vivo. Next, we presented a diagram to illustrate the mechanism of the circ_LDLR/miR-195-5p/LIPH axis in the regulation of PTC cell proliferation, migration, invasion and apoptosis (Fig. 7g).

## Discussion

Mounting evidence has shown that circRNAs are aberrantly expressed in tumors and involved in regulating tumor progression [6]. Herein, our goal was to investigate the functional effects of circ_LDLR on PTC. We results showed that circ_LDLR level was notably increased in PTC. Further functional analysis showed that circ_LDLR knockdown repressed PTC cell growth and motility and facilitated apoptosis. Moreover, circ_LDLR could regulate miR-195-5p/LIPH axis, thereby promoting PTC progression.

Up to date, several of circRNAs was revealed to be dysregulated and play an crucial role in promoting the malignant behaviors of PTC cells. For example, circ_0039411 was elevated in PTC and knockdown of circ_0039411 impeded cell proliferation and motility and accelerated apoptosis in PTC cells by regulating miR-1179 and miR-1205 [25]. Circ_0025033 was elevated in PTC and promoted PTC cell growth and metastasis and inhibited apoptosis via sponging miR-1231 and miR-1304 [26]. Jin et al. disclosed that circ_0004458 was elevated in PTC and silencing of circ_0004458 hampered cell growth and facilitated apoptosis and cell cycle arrest in PTC cells in vitro and restrained tumorigenesis in vivo [27]. These findings all indicated the oncogenic role of circRNAs in PTC. In this work, we investigated the functional roles of circ_LDLR in PTC for the first time. As a result, we found compared to corresponding normal tissues and cells, circ_LDLR level was raised in PTC tissues and cells. Functionally, cell growth and metastasis were drastically repressed and apoptosis was apparently induced in circ_LDLR silencing PTC cells. Previous studies have verified that the downregulation of Twist1 is related to induction of E-cadherin, and Twist1 inactivation suppressed tumor metastasis by regulating epithelial-mesenchymal transition (EMT) [28, 29]. Thus, we further determined the effect of circ_LDLR Twist1 and E-cadherin. Our data showed that Twist1 level was dropped and E-cadherin level was raised after circ_LDLR deficiency in PTC cells. Furthermore, xenograft experiments displayed that circ_LDLR knockdown hampered tumorigenesis of PTC in vivo. Our results demonstrated that circ_LDLR played a positive role in PTC.

In recent years, the effects of circRNA/miRNA/mRNA interactions on tumorigenesis have attracted the attentions of more and more researchers [30]. Herein, miR-195-5p was confirmed as a target of circ_LDLR. Wang et al. disclosed that miR-195 level was dropped in thyroid cancer and its elevation inhibited thyroid cancer cell growth by targeting Raf1 [31]. Yin et al. unraveled that miR-195 overexpression hampered cell growth and metastasis in PTC cells via interacting with CCND1 and FGF2 [17]. In this work, miR-195-5p was determined to be weakly expressed in PTC and negatively correlated with circ_LDLR expression. MiR-195-5p overexpression could impede PTC cell growth and motility and facilitated apoptosis. More importantly, inhibitors of miR-195-5p effectively ameliorated the suppressive impact of circ_LDLR silencing on PTC cell progression, indicating that circ_LDLR could regulate PTC progression by sponging miR-195-5p.

LIPH has been demonstrated to be increased in PTC, and LIPH knockdown suppressed PTC cell colony formation, proliferation and metastasis [21]. Here, we confirmed that LIPH was a target gene of miR-195-5p and high level of LIPH was observed in PTC. Furthermore, the inhibitory impacts of miR-195-5p overexpression on the behaviors of PTC cells were abrogated by LIPH elevation, indicating that miR-195-5p could intervene PTC development by targeting LIPH.

However, this study also possessed certained shortcomings. We only explored the underlying mechanism of circ_LDLR in regulating PTC cell progression in vitro. We will conduct experiments to investigate the mechanism of circ_LDLR in vivo in the future. Moreover, the downstream signaling of LIPH will be considered in our further study.

## Conclusion

Taken together, our research unraveled that circ_LDLR was upregulated in PTC and circ_LDLR overexpression promoted the oncogenic properties of PTC cells by modulating LIPH via sponging miR-195-5p. This study identified a novel network circ_LDLR/miR-195-5p/LIPH axis in promoting PTC development, which might provide fresh thought to explore the therapeutic targets for PTC.

## Supplementary information

**Additional file 1: Fig. S1.** The effect of circ_LDLR knockdown  on linear LDLR level in PTC cells. a, b The levels of circ_LDLR and linear LDLR mRNA in TPC-1 and SW579 cells  transfected with si-NC or si-circ_LDLR were determine dby qRT-PCR. *P < 0.05. Each bar represents mean ± SD.

**Additional file 2: Fig. S2.** Circ_LDLR promoted PTC cell colony formation, proliferation, migration and invasion and suppressed cell apoptosis. Circ_LDLR or Vector was transfected into TPC-1 and SW579 cells. a-d The expression levels of circ_LDLR, miR-195-5p, LIPH mRNA and LIPH protein in TPC-1 and SW579 cells were determined using qRT-PCR assay or western blot assay. e-g The colony formation and proliferation of TPC-1 and SW579 cells were determined by colony formation assay and MTT assay, respectively. h The protein level of Ki67 in TPC-1 and SW579 cells was measured by western blot assay. i The apoptosis of TPC-1 and SW579 cells was analyzed by flow cytometry analysis. j, k The migration and invasion of TPC-1 and SW579 cells were evaluated by transwell assay. l, m The protein levels of Twist1 and E-cadherin in TPC-1 and SW579 cells were measured by western blot assay. *P < 0.05. Each bar represents mean ± SD.

**Additional file 3: Fig. S3.** MiR-195-5p inhibition  promoted PTC cell progression. TPC-1 and SW579 cells were transfected with anti-miR-NC or anti-miR-195-5p. a The expression level of miR-195-5p in TPC-1 and SW579 cells was determined by qRT-PCR assay. b–d The colony formation and proliferation of TPC-1 and SW579 cells were evaluated by colony formation assay and MTT assay, respectively. e The protein level of Ki67 in TPC-1 and SW579 cells was measured by western blot assay. f The apoptosis of TPC-1 and SW579 cells was explored by flow cytometry analysis. g, h The migration and invasion of TPC-1 and SW579 cells were detected by transwell assay. i, j The protein levels of Twist1 and E-cadherin in TPC-1 and SW579 cells were measured via western blot assay. *P < 0.05. Each bar represents mean ± SD.

## Data Availability

The data sets used and/or analyzed during the current study are available from the corresponding author on reasonable request.

## References

[1] Lim H, Devesa SS, Sosa JA, Check D, Kitahara CM (2017). Trends in thyroid cancer incidence and mortality in the United States, 1974–2013. JAMA.

[2] Jiang C, Cheng T, Zheng X, Hong S, Liu S, Liu J, Wang J, Wang S (2018). Clinical behaviors of rare variants of papillary thyroid carcinoma are associated with survival: a population-level analysis. Cancer Manag Res..

[3] Ferlay J, Shin HR, Bray F, Forman D, Mathers C, Parkin DM (2010). Estimates of worldwide burden of cancer in 2008: GLOBOCAN 2008. Int J Cancer.

[4] Haugen BR, Alexander EK, Bible KC, Doherty GM, Mandel SJ, Nikiforov YE, Pacini F, Randolph GW, Sawka AM, Schlumberger M (2016). 2015 American Thyroid Association Management Guidelines for adult patients with thyroid nodules and differentiated thyroid cancer: The American Thyroid Association Guidelines Task Force on thyroid nodules and differentiated thyroid cancer. Thyroid..

[5] Qu S, Yang X, Li X, Wang J, Gao Y, Shang R, Sun W, Dou K, Li H (2015). Circular RNA: a new star of noncoding RNAs. Cancer Lett.

[6] Zhou R, Wu Y, Wang W, Su W, Liu Y, Wang Y, Fan C, Li X, Li G, Li Y (2018). Circular RNAs (circRNAs) in cancer. Cancer Lett.

[7] Zhou W, Wu G, Li J, Lin X, Sun Y, Xu H, Shi P, Gao L, Tian X (2020). circRASSF2 acts as ceRNA and promotes papillary thyroid carcinoma progression through miR-1178/TLR4 signaling pathway. Mol Ther Nucleic Acids..

[8] Wang M, Chen B, Ru Z, Cong L (2018). CircRNA circ-ITCH suppresses papillary thyroid cancer progression through miR-22-3p/CBL/beta-catenin pathway. Biochem Biophys Res Commun.

[9] Peng N, Shi L, Zhang Q, Hu Y, Wang N, Ye H (2017). Microarray profiling of circular RNAs in human papillary thyroid carcinoma. PLoS ONE.

[10] He L, Hannon GJ (2004). MicroRNAs: small RNAs with a big role in gene regulation. Nat Rev Genet.

[11] Chou CK, Chen RF, Chou FF, Chang HW, Chen YJ, Lee YF, Yang KD, Cheng JT, Huang CC, Liu RT (2010). miR-146b is highly expressed in adult papillary thyroid carcinomas with high risk features including extrathyroidal invasion and the BRAF(V600E) mutation. Thyroid..

[12] Ma Y, Qin H, Cui Y (2013). MiR-34a targets GAS1 to promote cell proliferation and inhibit apoptosis in papillary thyroid carcinoma via PI3K/Akt/Bad pathway. Biochem Biophys Res Commun.

[13] Liu L, Wang J, Li X, Ma J, Shi C, Zhu H, Xi Q, Zhang J, Zhao X, Gu M (2015). MiR-204-5p suppresses cell proliferation by inhibiting IGFBP5 in papillary thyroid carcinoma. Biochem Biophys Res Commun.

[14] Wu J, Ji A, Wang X, Zhu Y, Yu Y, Lin Y, Liu Y, Li S, Liang Z, Xu X (2015). MicroRNA-195-5p, a new regulator of Fra-1, suppresses the migration and invasion of prostate cancer cells. J Transl Med..

[15] Zhou S, Yu L, Xiong M, Dai G (2018). LncRNA SNHG12 promotes tumorigenesis and metastasis in osteosarcoma by upregulating Notch2 by sponging miR-195-5p. Biochem Biophys Res Commun.

[16] Li M, Ren CX, Zhang JM, Xin XY, Hua T, Wang HB, Wang HB (2018). The effects of miR-195-5p/MMP14 on proliferation and invasion of cervical carcinoma cells through TNF signaling pathway based on bioinformatics analysis of microarray profiling. Cell Physiol Biochem.

[17] Yin Y, Hong S, Yu S, Huang Y, Chen S, Liu Y, Zhang Q, Li Y, Xiao H (2017). MiR-195 inhibits tumor growth and metastasis in papillary thyroid carcinoma cell lines by targeting CCND1 and FGF2. Int J Endocrinol..

[18] Houben AJ, Moolenaar WH (2011). Autotaxin and LPA receptor signaling in cancer. Cancer Metastasis Rev.

[19] Lin ME, Herr DR, Chun J (2010). Lysophosphatidic acid (LPA) receptors: signaling properties and disease relevance. Prostaglandins Other Lipid Mediat.

[20] Ishimine H, Zhou R, Sumitomo K, Ito Y, Seki Y, Yoshida Y, Kurisaki A (2016). Lipase member H frequently overexpressed in human esophageal adenocarcinomas. Tumour Biol.

[21] Cui M, Jin H, Shi X, Qu G, Liu L, Ding X, Wang Y, Niu C (2014). Lipase member H is a novel secreted protein associated with a poor prognosis for breast cancer patients. Tumour Biol.

[22] Li Y, Zhou X, Zhang Q, Chen E, Sun Y, Ye D, Wang O, Zhang X, Lyu J (2019). Lipase member H is a downstream molecular target of hypoxia inducible factor-1alpha and promotes papillary thyroid carcinoma cell migration in BCPAP and KTC-1 cell lines. Cancer Manag Res..

[23] Minna E, Romeo P, Dugo M, Cecco LD, Todoerti K, Pilotti S, Perrone F, Seregni E, Agnelli L, Neri A, Greco A, Borrello MG (2016). MiR-451a is underexpressed and targets AKT/mTOR pathway in papillary thyroid carcinoma. Oncotarget..

[24] Schulten HJ, Al-Mansouri Z, Baghallab I, Bagatian N, Subhi O, Karim S, Al-Aradati H, Al-Mutawa A, Johary A, Meccawy AA (2015). Comparison of microarray expression profiles between follicular variant of papillary thyroid carcinomas and follicular adenomas of the thyroid. BMC Genomics..

[25] Yang Y, Ding L, Li Y, Xuan C (2020). Hsa_circ_0039411 promotes tumorigenesis and progression of papillary thyroid cancer by miR-1179/ABCA9 and miR-1205/MTA1 signaling pathways. J Cell Physiol.

[26] Pan Y, Xu T, Liu Y, Li W, Zhang W (2019). Upregulated circular RNA circ_0025033 promotes papillary thyroid cancer cell proliferation and invasion via sponging miR-1231 and miR-1304. Biochem Biophys Res Commun.

[27] Jin X, Wang Z, Pang W, Zhou J, Liang Y, Yang J, Yang L, Zhang Q (2018). Upregulated hsa_circ_0004458 contributes to progression of papillary thyroid carcinoma by inhibition of miR-885-5p and activation of RAC1. Med Sci Monit.

[28] Kwok WK, Ling MT, Lee TW, Lau TC, Zhou C, Zhang X, Chua CW, Chan KW, Chan FL, Glackin C (2005). Up-regulation of TWIST in prostate cancer and its implication as a therapeutic target. Cancer Res.

[29] Yang MH, Hsu DS, Wang HW, Wang HJ, Lan HY, Yang WH, Huang CH, Kao SY, Tzeng CH, Tai SK (2010). Bmi1 is essential in Twist1-induced epithelial-mesenchymal transition. Nat Cell Biol.

[30] Rong D, Sun H, Li Z, Liu S, Dong C, Fu K, Tang W, Cao H (2017). An emerging function of circRNA-miRNAs-mRNA axis in human diseases. Oncotarget..

[31] Wang F, Jiang C, Sun Q, Yan F, Wang L, Fu Z, Liu T, Hu F (2015). miR-195 is a key regulator of Raf1 in thyroid cancer. Onco Targets Ther..

