# Supplementary figures and images for: Circ_LDLR promoted the development of papillary thyroid carcinoma via regulating miR-195-5p/LIPH axis
Source: Cancer Cell Int. 2020 Jun 15;20:241. doi: 10.1186/s12935-020-01327-3 (PMC7296738; doi:10.1186/s12935-020-01327-3)

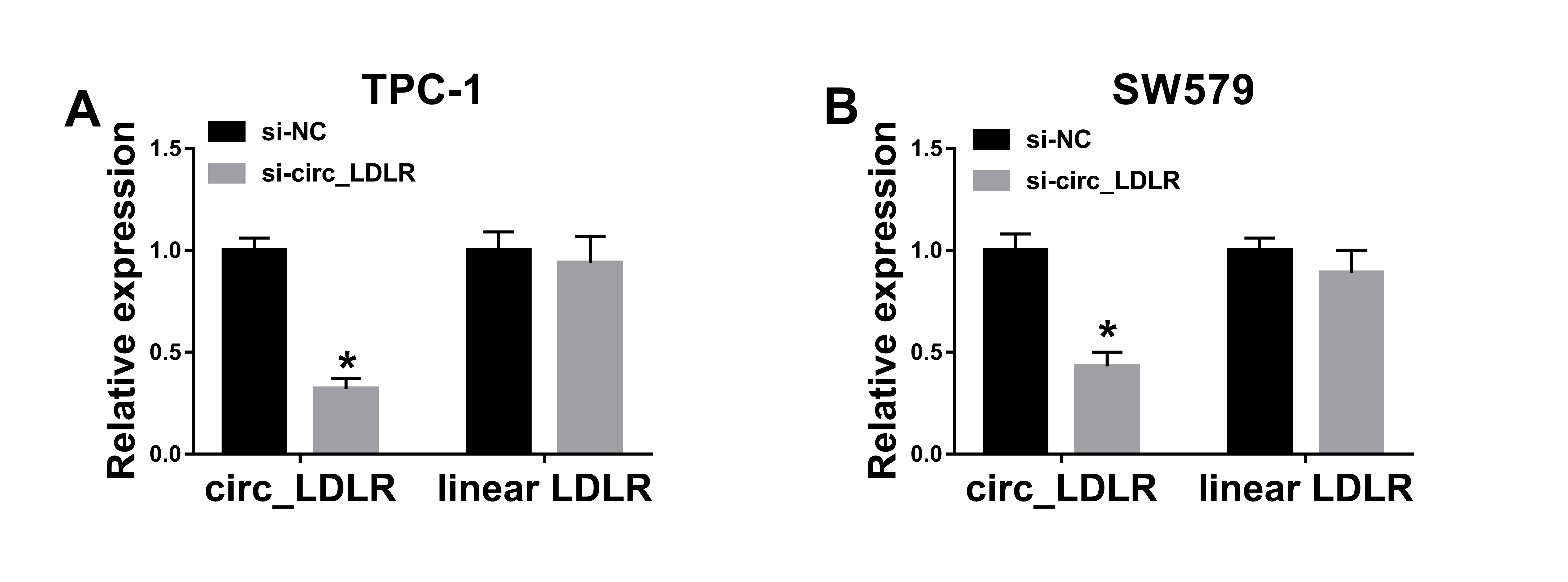

Supplement: Supplementary file 1 — Additional file 1: Fig. S1. The effect of circ_LDLR knockdown on linear LDLR level in PTC cells. a, b The levels of circ_LDLR and linear LDLR mRNA in TPC-1 and SW579 cells transfected with si-NC or si-circ_LDLR were determine dby qRT-PCR. *P < 0.05. Each bar represents mean ± SD. [file 12935_2020_1327_MOESM1_ESM.tif]

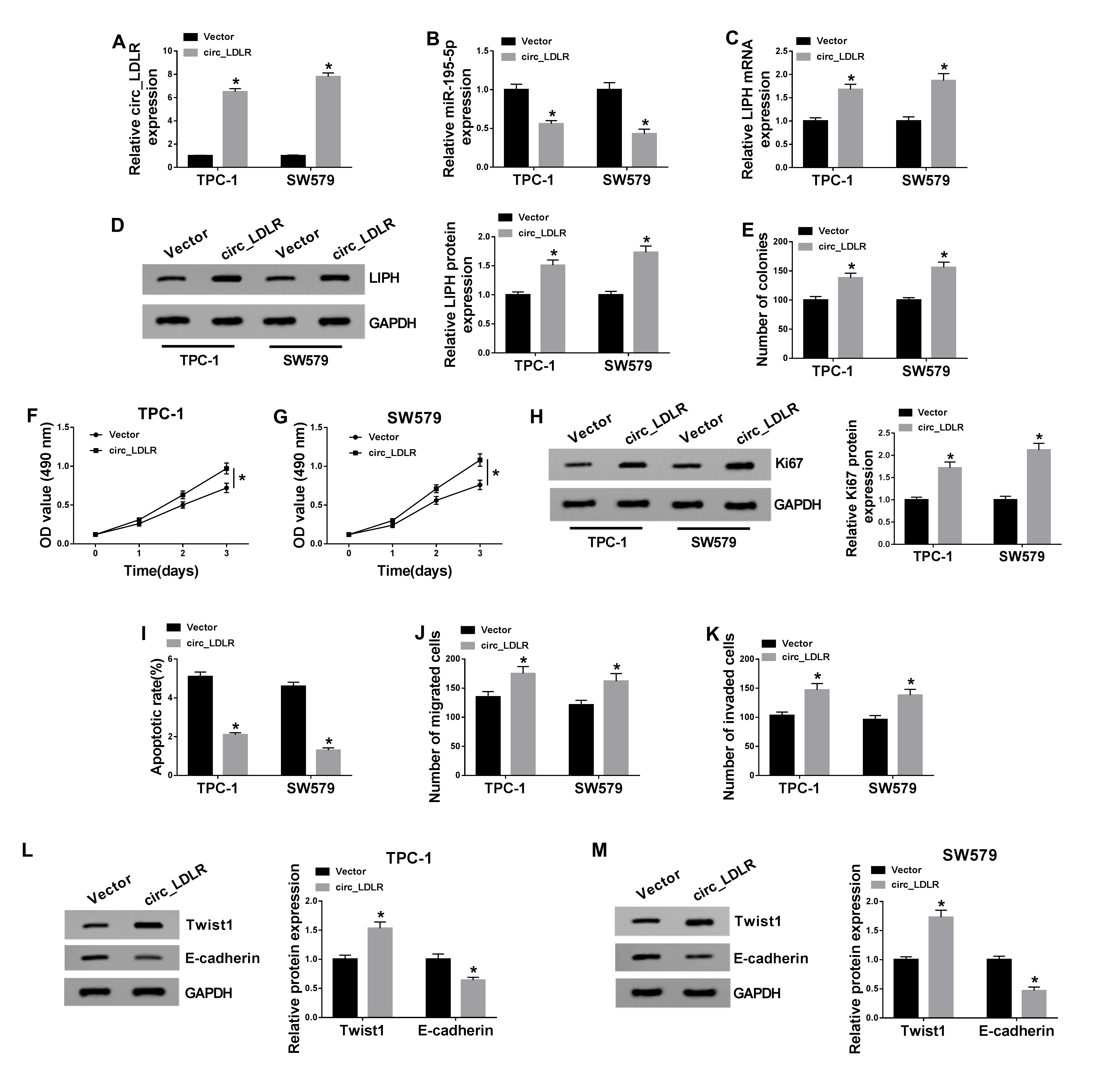

Supplement: Supplementary file 2 — Additional file 2: Fig. S2. Circ_LDLR promoted PTC cell colony formation, proliferation, migration and invasion and suppressed cell apoptosis. Circ_LDLR or Vector was transfected into TPC-1 and SW579 cells. a-d The expression levels of circ_LDLR, miR-195-5p, LIPH mRNA and LIPH protein in TPC-1 and SW579 cells were determined using qRT-PCR assay or western blot assay. e-g The colony formation and proliferation of TPC-1 and SW579 cells were determined by colony formation assay and MTT assay, respectively. h The protein level of Ki67 in TPC-1 and SW579 cells was measured by western blot assay. i The apoptosis of TPC-1 and SW579 cells was analyzed by flow cytometry analysis. j, k The migration and invasion of TPC-1 and SW579 cells were evaluated by transwell assay. l, m The protein levels of Twist1 and E-cadherin in TPC-1 and SW579 cells were measured by western blot assay. *P < 0.05. Each bar represents mean ± SD. [file 12935_2020_1327_MOESM2_ESM.tif]

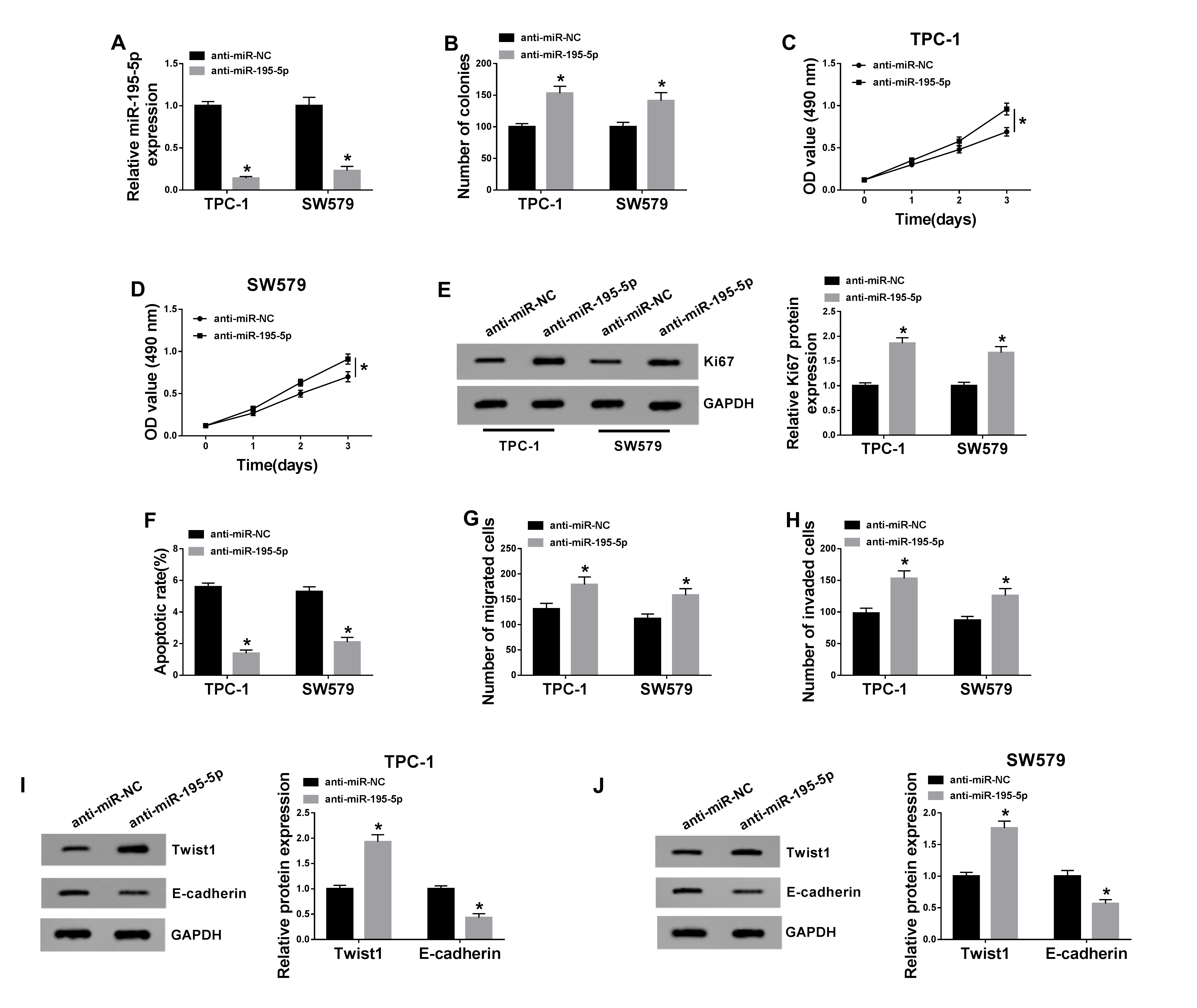

Supplement: Supplementary file 3 — Additional file 3: Fig. S3. MiR-195-5p inhibition promoted PTC cell progression. TPC-1 and SW579 cells were transfected with anti-miR-NC or anti-miR-195-5p. a The expression level of miR-195-5p in TPC-1 and SW579 cells was determined by qRT-PCR assay. b–d The colony formation and proliferation of TPC-1 and SW579 cells were evaluated by colony formation assay and MTT assay, respectively. e The protein level of Ki67 in TPC-1 and SW579 cells was measured by western blot assay. f The apoptosis of TPC-1 and SW579 cells was explored by flow cytometry analysis. g, h The migration and invasion of TPC-1 and SW579 cells were detected by transwell assay. i, j The protein levels of Twist1 and E-cadherin in TPC-1 and SW579 cells were measured via western blot assay. *P < 0.05. Each bar represents mean ± SD. [file 12935_2020_1327_MOESM3_ESM.tif]
